# Predictive factors for severe toxicity of sunitinib in unselected patients with advanced renal cell cancer

**DOI:** 10.1038/sj.bjc.6604456

**Published:** 2008-07-01

**Authors:** A A M van der Veldt, E Boven, H H Helgason, M van Wouwe, J Berkhof, G de Gast, H Mallo, C N Tillier, A J M van den Eertwegh, J B A G Haanen

**Affiliations:** 1 Department of Medical Oncology, VU University medical center, Amsterdam, The Netherlands; 2 Department of Medical Oncology, the Netherlands Cancer Institute, Antoni van Leeuwenhoek Hospital, Amsterdam, The Netherlands; 3 Epidemiology and Biostatistics, VU University medical center, Amsterdam, The Netherlands

**Keywords:** renal cell cancer, sunitinib, toxicity, dose reduction, non-clear cell histology

## Abstract

Sunitinib has been registered for the treatment of advanced renal cell cancer (RCC). As patient inclusion was highly selective in previous studies, experience with sunitinib in general oncological practice remains to be reported. We determined the efficacy and safety of sunitinib in patients with advanced RCC included in an expanded access programme. ECOG performance status >1, histology other than clear cell and presence of brain metastases were no exclusion criteria. Eighty-two patients were treated: 23% reached a partial response, 50% had stable disease, 20% progressed and six patients were not evaluable. Median progression-free survival (PFS) was 9 months and median overall survival (OS) was 15 months. Importantly, 47 patients (57%) needed a dose reduction, 35 (43%) because of treatment-related adverse events, 10 (12%) because of continuous dosing, and two because of both. Stomatitis, fatigue, hand–foot syndrome and a combination of grade 1–2 adverse events were the most frequent reasons for dose reduction. In 40 patients (49%), there was severe toxicity, defined as dose reduction or permanent discontinuation, which was highly correlated with low body surface area, high age and female gender. On the basis of age and gender, a model was developed that could predict the probability of severe toxicity.

Advanced renal cell cancer (RCC) has been recognised as a chemoresistant disease. The only treatment available has been cytokine-based therapy. Increasing knowledge of the underlying biology of RCC, and more specifically, the clear cell subtype, has recently changed the treatment options. Clear cell carcinomas, which account for 75% of all RCC subtypes, appear to contain an inactivated *von Hippel–Lindau (VHL)* tumour suppressor gene in at least 60% of these tumours (Brugarolas, 2007). *von Hippel–Lindau* gene alterations lead to elevated protein levels of hypoxia-induced factor-*α*, which upregulates vascular endothelial growth factor (VEGF) and platelet-derived growth factor (PDGF) genes and proteins (Brugarolas, 2007). The overexpression of these growth factors results in blood vessel formation which may account for the high vascular density of these tumours. Consequently, tumour angiogenesis has become an interesting therapeutic target in patients with metastatic RCC (mRCC).

Antiangiogenic agents, such as bevacizumab (Escudier *et al*, 2007b), sorafenib (BAY 43–9006) (Escudier *et al*, 2007a) and sunitinib (SU011248) (Motzer *et al*, 2007) have demonstrated significant antitumour activity in advanced RCC preferentially of the clear cell type excluding patients with poor prognosis. In a phase III clinical trial in mRCC, bevacizumab, a neutralising antibody against VEGF, in combination with interferon-*α* prolonged progression-free survival (PFS) with 4.8 months as compared to interferon-*α* alone (Escudier *et al*, 2007b). Sunitinib and sorafenib are oral tyrosine kinase inhibitors of the VEGF and PDGF receptors. In comparison with placebo, sorafenib prolonged PFS in cytokine-pretreated mRCC with almost 3 months (Escudier *et al*, 2007a). Sunitinib demonstrated a significantly prolonged PFS (11 *vs* 5 months) as well as a higher objective response rate than treatment with interferon-*α* (31 *vs* 6%) (Motzer *et al*, 2007). Temsirolimus, an inhibitor of mammalian target of rapamycin (mTOR) kinase, has demonstrated to improve the overall survival (OS) in RCC patients with unselected cancer histology and poor prognosis in comparison with interferon-*α* (11 months *vs* 7 and 8 months for, respectively, single-agent temsirolimus *vs* single-agent interferon-*α* and the combination) (Hudes *et al*, 2007).

In the pivotal trials on sunitinib, patients had to fulfil prespecified criteria. Eastern Cooperative Oncology Group (ECOG) performance status >1, brain metastases, uncontrolled hypertension or clinically significant cardiovascular events or disease during the preceding 12 months were exclusion criteria (Motzer *et al*, 2006a, 2006b, 2007). In addition, only patients with clear cell histology were allowed for entry in two out of the three previous studies (Motzer *et al*, 2006b, 2007). Nowadays, sunitinib can be prescribed widely to patients with advanced RCC, but the experience with this drug in an unselected patient population that does not meet the above-described criteria has yet to be revealed. Here, we report on a first experience with sunitinib treatment in a large advanced-stage RCC patient population reflecting general oncological practice and show that clinical benefit is comparable to that observed in the earlier phase II/III trials. An unexpectedly high number of patients, however, required dose reductions to maintain an acceptable quality of life.

## Patients and methods

### Patient population

From December 2005 to September 2006, patients with histologically confirmed advanced RCC were enrolled in a global expanded access programme (EAP) for treatment with sunitinib. Results are reported for patients treated in two centres in Amsterdam (VU University medical center and the Netherlands Cancer Institute). Until May 2006, patients were included only after cytokine-based therapy had failed and, thereafter, the drug was also available first-line. Inclusion criteria were as follows: age 18 years of age or older, adequate organ function (total serum bilirubin ⩽2 × upper limit of normal (ULN), serum transaminases <5 × ULN, serum creatinine ⩽2 × ULN, absolute neutrophil count ⩾1 × 10^9^ l^−1^, platelets ⩾75 × 10^9^ l^−1^, haemoglobin ⩾5.0 mmol l^−1^) and resolution of all toxic effects of prior systemic therapy, radiotherapy or surgical procedure according to National Cancer Institute-Common Toxicity Criteria (NCI-CTC) version 3.0 grade ⩽1. Before entry into the programme, each participant had to sign an institutional review board-approved protocol-specific informed consent in accordance with the national and institutional guidelines, which strictly adhere to the principles of the Declaration of Helsinki and its subsequent amendments.

Exclusion criteria were as follows: pregnancy or breast feeding, concurrent treatment in another therapeutic trial, previous treatment with sunitinib, congestive heart failure, myocardial infarction or coronary artery bypass graft in the previous 6 months, ongoing severe or unstable angina, any unstable arrhythmia requiring medication or another severe acute or chronic medical or psychiatric condition or laboratory abnormality that would make the patient inappropriate for entry in this EAP.

### Treatment, efficacy and adverse events

Sunitinib was administered orally at a dose of 50 mg daily, consisting of 4 weeks of treatment followed by a 2-week rest period in cycles of 6 weeks. A dose reduction of sunitinib (to 37.5 or 25 mg) was allowed depending on the type and severity of adverse events. If patients had symptoms of progressive disease (PD) during the rest period, there was the possibility for continuous dosing of sunitinib at 37.5 mg per day.

Patients underwent physical examination on day 1 of every cycle. Complete blood cell count and serum chemistry tests were carried out on day 1 and 28 of every treatment cycle. Complete blood cell count was also performed on day 14 of the first cycle. Electrocardiography was performed at baseline and on day 28 of the first treatment cycle. Haematological and non-haematological toxic effects were graded according to NCI-CTC version 3.0. Toxicity evaluation was conducted on day 1, 14 and 28 of the first treatment cycle and on day 1 and 28 of each treatment cycle thereafter. If grade 3 haematological toxicity was recorded, the treatment was withheld until the recovery grade ⩽2 or blood counts had returned to baseline after which sunitinib was resumed at the same dose level. In case of grade 4 haematological toxicity and grade 3 or 4 non-haematological toxicity, treatment was delayed until side effects had recovered to grade ⩽2 or grade 1, respectively, or had returned to baseline after which the dose was reduced by one level at the discretion of the treating physician. In the case of grade 4 non-haematological toxicity, treatment was discontinued.

Computed tomography (CT) or magnetic resonance imaging (MRI) was performed at baseline and every two to three cycles of treatment to assess clinical response according to Response Evaluation Criteria in Solid Tumours (RECIST) (Therasse *et al*, 2000).

### Data analysis

Specific case report forms were used for data entry. For response evaluation and toxicity, the cutoff date for data analysis was 1 March, 2007. For survival analysis, data collection was closed on 1 September, 2007. Patients were classified according to two prognostic classification systems for mRCC: (1) the Memorial Sloan–Kettering Cancer Center (MSKCC) prognostic criteria (based on five risk factors: low Karnofsky performance status (<80%), high lactate dehydrogenase (LDH, >1.5 times the ULN), low serum haemoglobin, high-corrected serum calcium (>10 mg per 100 ml) and time from initial diagnosis to treatment of less than 1 year) (Motzer *et al*, 2002) and (2) the prognostic criteria for VEGF-targeted therapy according to Choueiri *et al* (2007) (based on the following 5 risk factors: time from diagnosis to treatment <2 years, baseline platelet count >300 × 10^9^ l^−1^, baseline neutrophil count >4.5 × 10^9^ l^−1^, baseline corrected calcium <8.5 mg per 100 ml or >10 mg per 100 ml and initial ECOG performance status >0).

Efficacy parameters were best response, time-to-treatment failure (TTF), PFS and OS. The TTF was defined as the time between the first day of treatment and the date of the first event considered as failure of treatment. Such events could be disease progression, early discontinuation (owing to unacceptable toxicity, patient's request and lost to follow-up) or death. The PFS was the time between the first day of sunitinib and the date of PD on CT or MRI, clear clinical evidence of PD or death owing to PD within 12 weeks after the last response evaluation. If a patient had not progressed, PFS was censored at the time of the last follow-up. If the PD date was unknown or a patient died owing to PD later than 12 weeks after the last response evaluation, PFS was censored at the last adequate tumour assessment. Overall survival was the time between the first day of treatment and the date of death or the date at which patients were last known to be alive. Progression-free survival and OS were calculated with the Kaplan–Meier method.

Severe toxicity was defined as dose reduction or permanent discontinuation of sunitinib because of treatment-related adverse events.

The following clinical characteristics were analysed for a possible relation with severe toxicity: gender, age, body surface area (BSA), ECOG performance status, tumour type, presence of primary tumour, time of diagnosis to treatment, prior cytokine based-therapy, previous radiation therapy, number of tumour sites, liver metastases, MSKCC risk groups, Chouieri risk groups and baseline biochemical parameters. Baseline biochemical parameters (haemoglobin, LDH, albumin, creatinine, alkaline phosphatase and corrected calcium) were all quantified as a factor of the ULN. Statistical analysis was carried out using SPSS software (SPSS for Windows 15.0, SPSS, Inc., Chicago, IL, USA). Univariate logistic regression was performed to explore associations between the separate clinical characteristics and severe toxicity. Thereafter, the variables with a significance of *P*<0.05 were used for multivariate logistic regression analysis.

## Results

### Patients and treatment

Eighty-two patients with advanced RCC were registered in the EAP. Patient characteristics are depicted in Table 1. Fourteen patients had non-clear cell histology, 17 patients had a performance status of ECOG >1, 16 patients had a concurrent primary tumour *in situ* and five patients had concurrent brain metastases. All patients received sunitinib for a period of at least 1 week. At the time of the analysis, 18 patients were still on study and 64 had discontinued sunitinib. Reasons for termination were PD (*n*=44), adverse events either related to treatment or disease (*n*=14), early death (*n*=3), nephrectomy after downsizing of the primary tumour (*n*=2) and radiofrequency ablation of liver metastases (*n*=1).

### Efficacy

With respect to best response, 19 (23%) patients achieved a partial response (PR) (15 confirmed and four non-confirmed), 41 (50%) patients had stable disease (SD) and 16 (20%) patients had PD. Six patients could not be evaluated, five as a result of early termination and one patient because of bone metastases only. Impressive responses were observed in the 16 primary tumours (Van der Veldt *et al*, 2008) and six of them achieved a PR. Ten out of 14 patients with non-clear cell histology and two out of five patients with brain metastases had SD. No objective responses were observed in these subgroups. Seven patients developed symptomatic brain metastases as first or only sign of PD (Helgason *et al*, 2008).

Excluding the three patients with nephrectomy and radiofrequency ablation, the median TTF in 61 patients who discontinued treatment with sunitinib, was 3.6 months (range: 0.3–18.4 months). The median PFS (*n*=77) was 9.3 months (range: 0.5–18.3 months; Table 2). The patient with bone metastases only was included in PFS and had clinical benefit lasting >8 months. PFS was censored for the patient with radiofrequency ablation. The median PFS in the 14 patients with non-clear cell histology was 3.2 months (range: 1.2–17.0 months). The median OS for the total patient population was 15.0 months (range: 0.5–19.4 months; Table 2).

**Figure 1:**
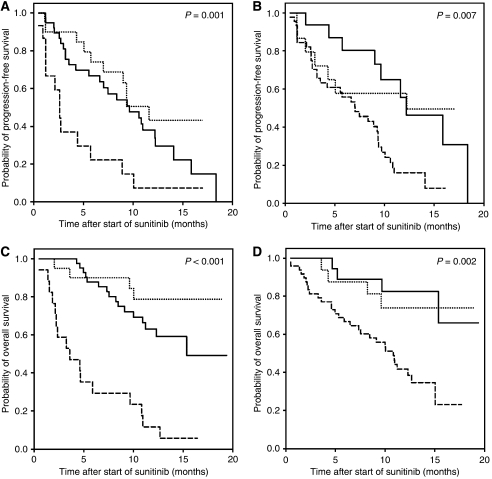
Kaplan–Meier curves for progression-free survival and overall survival of mRCC patients treated with sunitinib for risk groups 1 (…), 2 (—) and 3 (– –) according to the MSKCC criteria (Motzer *et al*, 2002) (**A** and **C**) and the criteria according to Choueiri *et al* (2007) (**B** and **D**).

Additional analyses were carried out with respect to individual patient characteristics and the course of the disease. Both the MSKCC criteria (Motzer *et al*, 2002) and the criteria according to Choueiri *et al* (2007) correctly predicted the PFS (*P*=0.001 and *P*=0.007, respectively) as well as the OS (*P*<0.001 and *P*=0.002, respectively; Table 2 and Figure 1). The predictive value of the number of disease sites was rather low, but for OS, the number of disease sites still had prognostic value (*P*=0.039).

### Safety and dose reductions

The most frequent non-haematological grade 1–2 adverse events were stomatitis, nausea, diarrhoea, vomiting, fatigue, hand–foot syndrome and taste alteration (Table 3). Grade 3 adverse events most frequently occurring were hand–foot syndrome (11%), stomatitis (9%), diarrhoea (7%), fatigue (6%) and hypertension (6%). Detailed information on three patients with reversible cognitive disorders grade 2–3 has been published elsewhere (Van der Veldt *et al*, 2007). Thrombocytopenia, leucocytopenia and neutropenia were the most frequently reported haematological adverse events (Table 4). The frequency of grade 3 haematological adverse events was also low, being lymphopenia (9%), thrombocytopenia (7%), neutropenia (7%), leucocytopenia (5%) and anaemia (1%). No grade 4 adverse events were observed.

In 40 (49%) patients, there was severe toxicity requiring dose reduction in 37 patients (median time to dose reduction 1.4 months, range: 0.2–12.4 months) and permanent discontinuation in three patients (for all three patients within 0.5 month; Table 5). Dose reduction in at least 6 out of 37 patients was not sufficient to alleviate symptoms, because of which sunitinib had to be discontinued. Stomatitis grade 3 was the most frequently reported reason for dose reduction, followed by fatigue, hand–foot syndrome and the combination of several grade 1–2 adverse events. In addition, 10 (12%) patients needed continuous dosing because of PD or recurrence of disease-related symptoms during the 2-week rest period (median time to continuous dosing 3.1 months, range: 1.1–11.8 months). Two patients had dose reduction because of both toxicity and continuous dosing (time to dose reduction 1.4 and 4.2 months).

**Figure 2:**
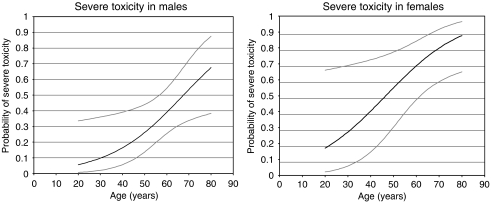
Probability of severe toxicity from sunitinib (50 mg per day 4 weeks on and 2 weeks off) in patients with advanced RCC based on the following model: Probability of severe toxicity in male patients=exp (−3.986+0.059^*^age)/(exp (−3.986+0.059^*^age)+1) Probability of severe toxicity in female patients=exp (−2.750+0.059^*^age)/(exp (−2.750+0.059^*^age)+1) Grey lines represent confidence intervals.

Female gender, high age, low BSA and to a lesser extent also high LDH were significantly related with severe toxicity (univariate logistic regression; *P*=0.006, *P*=0.006, *P*=0.005 and *P*=0.035, respectively). There was no significant relation between severe toxicity and the separate prognostic risk groups according to the MSKCC criteria (Motzer *et al*, 2002) as well as the criteria of Choueiri *et al* (2007). In multivariate logistic regression of gender, age, BSA and LDH, the latter two variables appeared to be of no additional significance in the prediction of severe toxicity. In multivariate logistic regression, gender and age had a significant effect (*P*=0.018 and *P*=0.024, respectively) and the combination of these two variables was highly predictive for severe toxicity (*P*=0.001). On the basis of gender and age, a model was developed to predict the probability of severe toxicity in male patients and female patients (Figure 2).

## Discussion

We here describe the efficacy and safety of sunitinib treatment in an unselected mRCC patient population as can be found in general oncological practice. In our mRCC patients, the SD rate (50%) resembled that observed in the large phase III clinical trial in which sunitinib was compared with interferon-*α*, but the PR rate was slightly less (23 *vs* 31%; Motzer *et al*, 2007). The PR rate in patients with clear cell histology (28%), however, was similar to that in the phase III study in patients with clear cell mRCC only (Motzer *et al*, 2007). The MSKCC risk groups (Motzer *et al*, 2002) appropriately predicted PFS and OS in this patient population, which indicates that the Motzer prognostic factors model is still valid to predict survival in mRCC in the sunitinib era. The prognostic criteria of Choueiri *et al* (2007) designed for patients with clear cell histology receiving VEGF-targeted therapy, however, did not discriminate a difference in OS between risk groups 1 and 2 in our patient population. An explanation may be that we have treated a large number of cytokine-pretreated patients (65%) as well as patients with non-clear cell histology (17%).

In the non-clear cell histology patient population, 10 out of 14 patients had SD, whereas no PR was observed. Recently, Choueiri *et al* (2008) have reported their experience with sunitinib and sorafenib in patients with non-clear cell histology. During either sunitinib or sorafenib treatment, 5 out of 53 patients with non-clear cell histology, either papillary or chromophobe tumours, reached a PR, whereas 36 patients had SD of more than 3 months. The present data and the study of Choueiri *et al* (2008) indicate that patients with non-clear cell histology may benefit from sunitinib. Furthermore, patients with poor performance status (ECOG >1) and brain metastases also experienced benefit from sunitinib treatment in 65 and 40% of cases, respectively.

Treatment-related adverse events were mostly grade 1 or 2 and only few grade 3 toxicities were observed. The incidence rates of the most common grade 3 adverse events requiring dose discontinuation and/or reduction, such as hand–foot syndrome, stomatitis, diarrhoea, fatigue and hypertension were grossly similar to the rates reported in previous trials (Demetri *et al*, 2006; Motzer *et al*, 2006a, 2006b, 2007). In this patient population, we observed a relatively lower incidence of thrombocytopenia and leucocytopenia than that reported in the largest trial on sunitinib so far (Motzer *et al*, 2007). Although thyroid function was not measured consistently, only five patients experienced hypothyroidism grade 1–2 (data not shown).

More than half of our patients needed a dose reduction of sunitinib and 35 out of 82 patients (43%) because of treatment-related adverse events. In comparison, only for 32% of the patients treated with sunitinib in the large randomised phase III trial of sunitinib *vs* IFN-*α* a dose reduction was reported, which might partially be explained by a higher number of patients with ECOG ⩾1 in our population. The remarkably high number of dose reductions, however, was not only based on grade 3 toxicities, but also on the accumulation of a series of grade 1 and 2 adverse events. These toxicities were palliated in every possible way. Some adverse events, however, interfered excessively with daily life, such as stomatitis and taste alteration requiring changes in food habits, hand–foot syndrome limiting walking and the urgent pattern of diarrhoea with risk for soiling. In this respect, the NCI-CTC grading system is inadequate to express the impact of particular toxicities of sunitinib for the well-being of the patient.

Our findings are indicative that the sunitinib dosing schedule is not optimal for unselected mRCC patients and that a number of patients are initially overtreated resulting in unnecessary adverse events. On the other hand, patients who do not experience any toxicity may be undertreated. Therefore, dosing on the basis of BSA might be meaningful, as BSA was highly correlated with severe toxicity. In the previous phase I study in patients with solid tumours, the simulated intrapatient variability in drug plasma levels between BSA-normalised and fixed dosing was comparable on days 1 and 28 for both sunitinib and its major plasma metabolite SU012622 (Faivre *et al*, 2006). It was concluded that no or minimal improvement in variability could be expected from calculating the dose on the basis of BSA. With respect to our data, it should be reconsidered to administer initial doses on the basis of BSA and taper off to tolerable doses if required, or increase the dose if no toxicity is observed. Alternatively, population-based sunitinib+SU012622 plasma levels could be of help to develop better algorithms for optimal sunitinib dosing.

With the use of the fixed dosing regimen, we not only found a highly significant correlation between severe sunitinib-related toxicity and patient characteristics BSA, but also with female gender and high age. We developed a model to predict the probability of severe toxicity based on gender and age in which BSA was not additive. Although the model requires external validation, it might be helpful to closely monitor patients at risk to develop invalidating adverse events on sunitinib given in the currently proposed schedule. It can also be proposed to dose patients on BSA and determine, whether female gender and high age remain prognostic factors for severe toxicity. Any grade 3 toxicity was also significantly related to gender and BSA, but not to age. The occurrence of any grade 3 adverse event was the reason for dose reduction or discontinuation of sunitinib in 79% of these patients.

Ten (12%) patients required continuous dosing at a lower dose of 37.5 mg daily owing to objective disease progression or recurrence of disease-related symptoms in the 2-week period of rest of the treatment cycle. Two phase II studies have demonstrated that the safety of a continuous dosing schedule of 37.5 mg per day in patients with RCC and gastrointestinal stromal tumours (GIST) was similar to that of the intermittent schedule (George *et al*, 2007; Srinivas *et al*, 2007). In addition, preliminary results suggest a comparable PFS and OS for the two dosing schedules (Faivre *et al*, 2007), although the objective response rate appears to be lower. In mRCC, the 4 weeks on and 2 weeks off schedule is the most preferred as a direct relation between the exposure to sunitinib (area under the plasma concentration-time curve), which is the highest during the 4 weeks on 50 mg per day, and a higher probability of PR, longer time-to-tumour progression, longer OS and greater decrease in tumour volume have been observed (Houk *et al*, 2007).

In conclusion, sunitinib demonstrates clinical benefit in unselected mRCC patients, including patients with non-clear cell histology, brain metastases and an ECOG performance status >1. The need for dose reduction owing to adverse events in this unselected mRCC patient population is rather high. Gender, age and BSA are highly predictive of severe toxicity. Attempts to optimise the dosing schedule of sunitinib in unselected mRCC patients are warranted.

## Figures and Tables

**Table 1:** Patient characteristics

	**Total *n*=82**
**Variable**	***n* (%)**
*Sex*	
Male	55 (67)
Female	27 (33)
	
Median age, years (range)	60 (25–84)
	
*ECOG performance status*	
0	33 (40)
1	29 (35)
2	12 (15)
3	5 (6)
Unknown	3 (4)
	
*Tumour type*	
Clear cell	68 (83)
Other	14 (17)
	
Previous nephrectomy	66 (80)
	
*Prior treatment*	
None	26 (32)
Cytokine based-therapy	53 (65)
Antiangiogenic therapy	5 (6)
	
Previous radiation therapy	25 (30)
	
*Number of disease sites*	
1	11 (13)
2	31 (38)
⩾3	40 (49)
	
*Sites of disease*	
Lung	66 (80)
Lymph nodes	43 (52)
Bone	26 (32)
Liver	22 (27)
Local recurrence	10 (12)
Brain	5 (6)
	
*MSKCC risk groups* a	
0 (favourable)	20 (24)
1–2 (intermediate)	41 (50)
⩾3 (poor)	17 (21)
Unknown	4 (5)
	
*Chouieri risk groups* b	
1 (0 or 1 adverse prognostic factor)	16 (20)
2 (2 adverse prognostic factors)	18 (22)
3 (>2 adverse prognostic factors)	48 (59)

ECOG=Eastern Cooperative Oncology Group; LDH=lactate dehydrogenase; MSKCC=Memorial Sloan–Kettering Cancer Center; VEGF=vascular endothelial growth factor.

a Risk groups according to MSKCC prognostic criteria (based on the five risk factors: low Karnofsky performance status (<80%), high LDH (>1.5 times the upper limit of normal), low serum haemoglobin, high-corrected serum calcium (>10 mg per 100 ml) and time from initial diagnosis to treatment of less than 1 year; Motzer *et al*, 2002).

b Prognostic risk groups for VEGF-targeted therapy according to Chouieri *et al* (2007) (based on the five risk factors: time from diagnosis to treatment <2 years, baseline platelet count >300 × 10^9^ l^−1^, baseline neutrophil count >4.5 × 10^9^ l^−1^, baseline corrected calcium <8.5 mg per 100 ml or >10 mg per 100 ml and initial ECOG performance status >0).

**Table 2:** Best tumour response, progression-free survival and overall survival

	**Best tumour response**					
	**PR**	**SD**	**PD**	**NE**	**Median PFS^a^**	**Median OS^a^**
**Variable**	***n* (%)**	***n* (%)**	***n* (%)**	***n* (%)**	**Months (range)**	**Months (range)**
All patients	19 (23)	41 (50)	16 (20)	6 (7)	9.3 (0.5–18.3)	15.0 (0.5–19.4)
						
*Histology*					*P*=0.528	*P*=0.105
Clear cell histology	19 (23)	31 (38)	12 (15)	6 (7)	9.3 (0.5–18.3)	15.0 (0.5–19.4)
Non-clear cell histology	0 (0)	10 (12)	4 (5)	0 (0)	3.2 (1.2–17.0)	6.5 (1.4–18.4)
						
*ECOG performance status*					*P*=0.397	*P*=0.049
ECOG ⩽1	14 (17)	34 (41)	12 (15)	2 (2)	9.4 (1.2–17.0)	NR (1.8–18.9)
ECOG >1	5 (6)	6 (7)	3 (4)	3 (4)	8.9 (0.5–18.3)	9.7 (0.5–19.4)
Unknown	0 (0)	1 (1)	1 (1)	1 (1)	1.2 (1.2–13.2)	2.2 (0.5–13.2)
						
*MSKCC risk groups* b					*P*=0.001	*P*<0.001
0 (favourable)	6 (7)	11 (13)	2 (2)	1 (1)	11.6 (1.1–17.0)	NR (2.0–18.9)
1–2 (intermediate)	9 (11)	23 (28)	7 (9)	2 (2)	9.6 (1.2–18.3)	15.4 (4.3–19.4)
⩾3 (poor)	2 (2)	6 (7)	7 (9)	2 (2)	2.6 (0.5–17.0)	3.6 (0.5–16.5)
Unknown	2 (2)	1 (1)	0 (0)	1 (1)	9.7 (9.3–13.2)	15.0 (0.5–15.0)
						
*Choueiri risk groups* c					*P*=0.007	*P*=0.002
1 (0 or 1 adverse prognostic factor)	3 (4)	8 (10)	4 (5)	1 (1)	12.2 (1.2–17.0)	NR (3.6–18.9)
2 (2 adverse prognostic factors)	8 (10)	8 (10)	1 (1)	1 (1)	12.2 (2.1–18.3)	NR (4.7–19.4)
3 (>2 adverse prognostic factors)	8 (10)	25 (30)	11 (13)	4 (5)	7.0 (0.5–16.1)	10.8 (0.5–17.7)
						
*Number of disease sites*					*P*=0.096	*P*=0.039
1	1 (1)	7 (9)	2 (2)	1 (1)	NR (1.2–17.0)	NR (3.6–17.2)
2	11 (9)	13 (16)	5 (6)	2 (2)	9.7 (0.9–18.3)	NR (1.5–19.4)
⩾3	7 (9)	21 (26)	9 (11)	3 (4)	8.4 (0.5–16.1)	11.0 (0.5–18.4)
						
*Miscellaneous*						
Concurrent primary tumour	6 (7)	6 (7)	4 (5)	0 (0)	9.3 (0.9–16.1)	15.0 (1.4–17.7)
Concurrent brain metastases	0 (0)	2 (2)	2 (2)	1 (1)	3.0 (2.6–12.2)	7.5 (3.6–18.4)
Previous cytokine-based therapy	14 (17)	27 (33)	8 (10)	4 (5)	10.6 (1.2–18.3)	NR (0.5–19.4)
Previous antiangiogenic therapy	1 (1)	1 (1)	2 (2)	1 (1)	2.6 (1.2–18.3)	4.6 (3.6–19.4)

ECOG=Eastern Cooperative Oncology Group; LDH=lactate dehydrogenase; MSKCC=Memorial Sloan–Kettering Cancer Center; NE=not evaluable; NR=median not reached; OS=overall survival; PD=progressive disease; PFS=progression-free survival; PR=partial response; SD=stable disease; VEGF=vascular endothelial growth factor.

a Median PFS and OS were calculated with the Kaplan–Meier method.

b Risk groups according to MSKCC prognostic criteria (based on the five risk factors: low Karnofsky performance status (<80%), high LDH (>1.5 times the upper limit of normal), low serum haemoglobin, high-corrected serum calcium (>10 mg per 100 ml) and time from initial diagnosis to treatment of less than 1 year; Motzer *et al*, 2002).

c Prognostic risk groups for VEGF-targeted therapy according to Chouieri *et al* (2007) (based on the five risk factors: time from diagnosis to treatment <2 years, baseline platelet count >300 × 10^9^ l^−1^, baseline neutrophil count >4.5 × 10^9^ l^−1^, baseline corrected calcium <8.5 mg per 100 ml or >10 mg per 100 ml and initial ECOG performance status >0).

**Table 3:** Non-haematological adverse events

**Non-haematological adverse event^a^**	**Grade 1 *n* (%)**	**Grade 2 *n* (%)**	**Grade 3 *n* (%)**	**All %**
Stomatitis	34 (41)	17 (21)	7 (9)	71
Nausea	31 (38)	9 (11)	4 (5)	54
Diarrhoea	27 (33)	8 (10)	6 (7)	50
Hand-foot syndrome	16 (20)	9 (11)	9 (11)	41
Fatigue	12 (15)	14 (17)	5 (6)	38
Vomiting	22 (27)	5 (6)	0 (0)	33
Taste alteration	20 (24)	6 (7)	0 (0)	32
Hypertension	5 (6)	9 (11)	5 (6)	23
Anorexia	6 (7)	12 (15)	0 (0)	22
Headache	7 (9)	6 (7)	2 (2)	18
Yellow skin	12 (15)	0 (0)	0 (0)	15
Rash/desquamation	8 (10)	4 (5)	0 (0)	15
Fever	7 (9)	4 (5)	0 (0)	13
Heartburn	7 (9)	4 (5)	0 (0)	13
Pain extremity	7 (9)	2 (2)	0 (0)	13
Esophagitis	5 (6)	3 (4)	1 (1)	11
Gastric complaints	7 (9)	2 (2)	0 (0)	11
Myalgia	8 (10)	1 (1)	0 (0)	11
Periorbital oedema	9 (11)	0 (0)	0 (0)	11
Dizziness	7 (9)	1 (1)	0 (0)	10
Epistaxis	8 (10)	0 (0)	0 (0)	10
Oedema	3 (4)	2 (2)	1 (1)	7
Pain mouth	2 (2)	1 (1)	1 (1)	5
Muscle weakness	0 (0)	2 (2)	1 (1)	4
Cognitive disorder	0 (0)	2 (2)	1 (1)	4
Hyperthyroidism	0 (0)	0 (0)	1 (1)	1
Transient ischaemic attack	0 (0)	0 (0)	1 (1)	1

a Adverse events grade 1 and 2 occurring in at least 10% of patients and all grade 3 events.

**Table 4:** Haematological adverse events

**Haematological adverse event**	**Grade 1 *n* (%)**	**Grade 2 *n* (%)**	**Grade 3 *n* (%)**	**All %**
Thrombocytopenia	27 (33)	7(9)	6 (7)	49
Leucocytopenia	17 (21)	16 (20)	4 (5)	45
Neutropenia	7 (9)	13 (16)	6 (7)	32
Lymphopenia	7 (9)	7 (9)	7 (9)	26
Anaemia	10 (12)	9 (11)	1 (1)	24

**Table 5:** Severe toxicity causing change of sunitinib dosing

**Reasons for dose reduction**	***n* (=37)**
*Non-haematological*	
Stomatitis grade 3	6
Fatigue grade 3	5
Hand-foot syndrome grade 2–3	5
Combination of several grade 1–2 toxicities	5
Diarrhoea grade 3	2
Cognitive disorder grade 2–3	2
Esophagitis grade 3	1
Headache grade 3	1
Hypertension grade 3	1
Pain mouth grade 3	1
Transient ischaemic attack grade 3	1
	
*Haematological*	
Neutropenia grade 3	3
Thrombocytopenia grade 3	3
Leucocytopenia grade 3	1
	
*Reasons for permanent discontinuation at once*	*n* (=3)
Cognitive disorder grade 2	1
Hypertension grade 3	1
Stomatitis grade 2	1
